# Chronic alcohol consumption alters extracellular space geometry and transmitter diffusion in the brain

**DOI:** 10.1126/sciadv.aba0154

**Published:** 2020-06-24

**Authors:** Silvia De Santis, Alejandro Cosa-Linan, Raquel Garcia-Hernandez, Lesia Dmytrenko, Lydia Vargova, Ivan Vorisek, Serena Stopponi, Patrick Bach, Peter Kirsch, Falk Kiefer, Roberto Ciccocioppo, Eva Sykova, David Moratal, Wolfgang H. Sommer, Santiago Canals

**Affiliations:** 1 Instituto de Neurociencias, Consejo Superior de Investigaciones Científicas and Universidad Miguel Hernández, Sant Joan d’Alacant, Spain.; 2 Department of Psychopharmacology, Central Institute of Mental Health, University of Heidelberg, 68159 Mannheim, Germany.; 3 Institute of Experimental Medicine AS CR, 142 20 Prague 4, Czech Republic.; 4 Charles University, 2nd Faculty of Medicine, 150 06 Prague 5, Czech Republic.; 5 School of Pharmacy, University of Camerino, Camerino, Italy.; 6 Department of Addiction Medicine, Central Institute of Mental Health, University of Heidelberg, 68159 Mannheim, Germany.; 7 Department of Clinical Psychology, Central Institute of Mental Health, University of Heidelberg, 68159 Mannheim, Germany.; 8 Institute of Neuroimmunology, Slovak Academy of Sciences, Bratislava, Slovakia.; 9 Center for Biomaterials and Tissue Engineering, Universitat Politècnica de València, Valencia, Spain.

## Abstract

Already moderate alcohol consumption has detrimental long-term effects on brain function. However, how alcohol produces its potent addictive effects despite being a weak reinforcer is a poorly understood conundrum that likely hampers the development of successful interventions to limit heavy drinking. In this translational study, we demonstrate widespread increased mean diffusivity in the brain gray matter of chronically drinking humans and rats. These alterations appear soon after drinking initiation in rats, persist into early abstinence in both species, and are associated with a robust decrease in extracellular space tortuosity explained by a microglial reaction. Mathematical modeling of the diffusivity changes unveils an increased spatial reach of extrasynaptically released transmitters like dopamine that may contribute to alcohol’s progressively enhanced addictive potency.

## Teaser

The alcohol-induced inflammatory response increases the diffusion range of small molecules in the extracellular brain parenchyma.

## INTRODUCTION

Alcoholic beverages are one of the most important commodities, with nearly 2 billion consumers worldwide. On the other side, alcohol use accounts for a substantial proportion of global health burden. In industrialized countries, about 10% of the disability-adjusted life years lost is due to the consumption of alcohol (*1*). Recent studies show that even very moderate drinking below the new stricter alcohol guidance in the United Kingdom does not prevent alcohol’s long-term adverse effects on brain function (*2*). Alcohol has a strong ability to induce neuroadaptations that promote its incentive salience, formation of strong consumption habits, and addictive behaviors, often leading to the development of alcohol use disorder (AUD). How alcohol produces its potent addictive effects despite being a weak reinforcer is a conundrum that is poorly understood and that likely hampers the development of successful interventions to limit heavy drinking.

Diffusion tensor magnetic resonance imaging (MRI), by measuring the diffusion of water in the brain parenchyma, is capable of detecting microstructural brain abnormalities following excessive alcohol consumption even before macrostructural (i.e., volumetric) changes appear (*3*). Our group recently used diffusion tensor imaging (DTI) to demonstrate convergent evidences of white matter alterations in humans with AUD and alcohol-exposed rats (*4*). While DTI is most commonly used in the study of white matter, it can be equally applied to the study of brain gray matter as well. Using an established rodent model of excessive alcohol intake, the Marchigian-Sardinian alcohol-preferring (msP) rats (*5*), a recent study suggested that the mean diffusivity (MD) measured by DTI in the whole brain is the most relevant parameter to characterize alcohol-induced states (*6*). No DTI studies have characterized MD in human alcoholics. MD measures the average displacement of water molecules in the tissue and, at low-diffusion weighting, particularly reflects extracellular water dynamics. Alterations in MD have been associated with neuroinflammatory processes both theoretically (*4*) and experimentally, using histological markers (*7*). Notably, neuroinflammation has been proposed as a mechanism of alcohol-related brain damage (*8*). However, diffusion parameters from DTI, while sensitive to changes in the extracellular space (ECS) geometry, do not have the necessary specificity to measure specific characteristics like the tortuosity and the extracellular volume fraction (*9*), necessary to describe neurotransmitter diffusion in the ECS. Instead, such parameters can be measured from direct invasive methods such as real-time iontophoresis, a point-source paradigm that uses tetramethylammonium as a small inert molecular marker (*10*–*12*).

The fact that alcohol can alter diffusion in gray matter ECS appears important for the pharmacodynamic effects of alcohol, because its rewarding effects, which vary strongly along the trajectory of AUD, are known to be mediated by glutamate, monoamine, and peptide neurotransmitters (*13*–*15*). These neuromodulators act partially via extrasynaptically located receptors that are reached via long-range diffusion across the ECS, constituting a hormone-like signaling mechanism in the brain, also called volume transmission (*16*). An alteration in the ECS is therefore expected to affect the dynamics of these neurotransmitters. To our knowledge, the possibility that chronic alcohol intake could affect the diffusion range of locally released neurotransmitters such as dopamine has not been considered so far.

Here, we use a multilevel experimental strategy, including (i) noninvasive DTI in msP rats before, during, and after alcohol drinking, as well as in patients with AUD and healthy controls; (ii) invasive in vivo real-time iontophoresis in the gray matter of chronic alcohol-drinking and naïve msP rats; (iii) postmortem assessment of extracellular matrix proteins and glial markers by quantitative immunohistochemistry; (iv) direct interference with the microglial pool in rats to demonstrate their sufficiency in the observed DTI phenomena; and (v) a numerical approach to solve the diffusion equation for dopamine in a healthy and alcohol-exposed ECS. Our combined results indicate widespread increased diffusivity in the gray matter of both alcohol-drinking animals and patients with AUD, which persists into early abstinence. In rats, this increase in gray matter MD is accompanied by the reduced tortuosity of the ECS and is linked to a microglial reaction. Modeling dopamine diffusivity in the ECS (*17*), we show that chronic alcohol consumption increases neurotransmitter concentrations over time. Therefore, we speculate that increased diffusivity in the ECS might be a new alcohol mechanism for addiction by potentiating dopamine’s far-reaching effects.

## RESULTS

### Chronic ethanol consumption increases MD in brain parenchyma in rats and humans

**Fig. 1:**
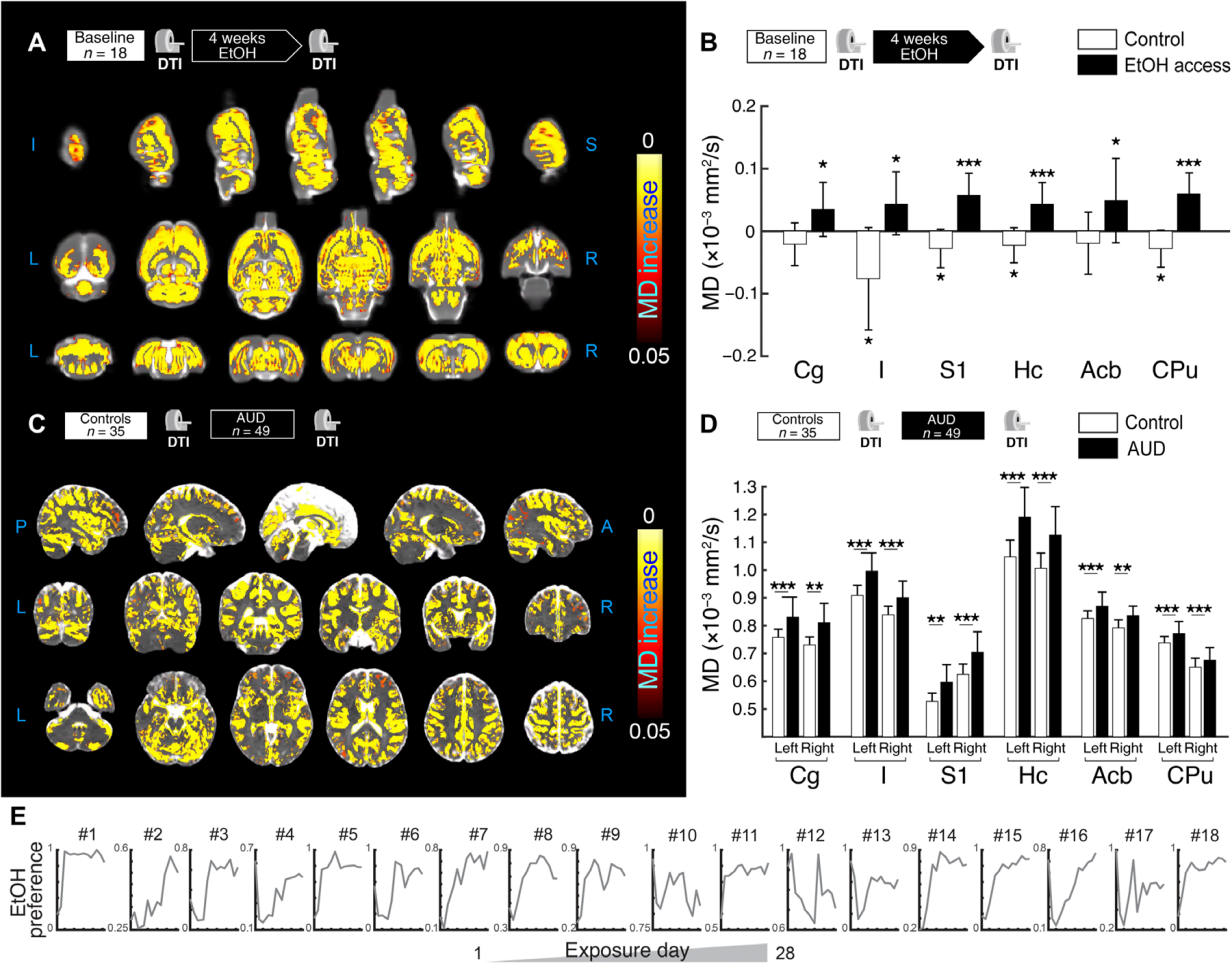
Effects of alcohol consumption in MD for rats and humans. (**A**) Voxel-wise statistical analysis showing longitudinal MD differences between rats at baseline and after 4 weeks of alcohol drinking. (**B**) Mean MD change versus baseline in six gray matter regions of interest (ROIs) for alcohol-drinking and control msP rats. Reported post hoc statistic shows significant differences between alcohol and baseline, as well as a significant age effect in the control group. (**C**) Voxel-wise statistical analysis showing cross-sectional MD differences between controls and alcohol-dependent patients at first scan. (**D**) Mean MD values in selected ROIs for controls and patients with AUD at first scan. Reported statistics show cross-sectional differences between controls and patients with AUD. For all panels, *P < 0.05, **P < 0.01, and ***P < 0.001. (**E**) Individual EtOH preference, measured as the ratio of EtOH/water-drinking amount, plotted as a function of the exposure day (from 1 to 28 days) in one batch of representative animals. Cg, cingulate cortex; I, insular cortex; S1, primary somatosensory cortex; Hc, hippocampus; Acb, accumbens; CPu, caudate-putamen.

We compared voxel-wise MD in rats before and after 4 weeks of alcohol drinking and nondrinking controls in a 2 × 2 within-subject design. In this period, alcohol consumption increased from 3 to 4 g kg^−1^ day^−1^ in the first 5 days to 7 to 8 g kg^−1^ day^−1^ from the10th day onward (individual daily EtOH consumption is reported in Fig. 1E). These drinking levels lead to pharmacologically relevant blood alcohol levels (BALs) as high as 1 g/liter in msP rats (*5*). In humans, these BALs are generally reached after consumption of four standard drinks in 1 hour, which is a legally intoxicating dose. Under these conditions, we observed an alcohol-induced brain-wide MD increase in the gray mater (Fig. 1A). Analysis by regions of interest (ROIs) confirmed the MD change (Fig. 1B and table S1). We found a significant group × time interaction effect (*P* < 10^−3^) across all anatomical regions evaluated (table S1). In four of six ROIs, we observed a decrease in MD due to the age effect (3 ± 0.9% reduction), consistent with the decreased water diffusivity in the brain with age (*18*), which contrasted against the alcohol-induced overall increase in MD (5 ± 0.3% increase) observed in all ROIs in the same time interval (Fig. 1B).

In humans, we found a pattern of changes similar to those found in rats. A widespread MD increase is observed under alcohol conditions compared to controls (Fig. 1C) in the voxel-wise analysis. The ROI-wise analysis confirmed that there is a significant overall increase in MD for AUD compared to controls [*F*(11,891) = 8.7, *P* < 0.001, and a mean effect size of 8.4%]. While more patients with AUD were smokers compared to controls, in light of the similarity between rat and human data, the difference in smoking is not likely to account for the observed MD differences. Also, the AUD cohort was slightly older than controls. While this was taken into account in the statistics, we repeated the analysis in an age-matched subset of subjects (fig. S1), finding no difference with the analysis performed in the full cohort. For detailed statistics and clinical descriptors, see table S2 and Table 1, respectively. We found no differences between alcohol and abstinence stages in both species [*F*(1,8) = 1.4, *P* = 0.269 for rats; *F*(1,49) = 0.9, *P* = 0.349 for humans; see fig. S2, A and B].

**Table 1:** Demographic and clinical data for healthy controls and patients. ADS, Alcohol Dependence Scale; FTND, Fagerstroem Test for Nicotine Dependence.

**Patient group, mean (SE)**				
	**Control (*n* = 35)**	**AUD cohort (*n* = 49)**	**Statistics**	***P* value**
Demographical variables				
Age, mean (SE), year	**41.8 (1.6)**	**47.5 (1.4)**	***t***_**82**_ **= 2.636**	**0.010**
Educational attainment, no. of participants				
No graduation	**0**	**1**	**Χ^2^_3_ = 9.054**	**0.029**
Primary school	**5**	**16**		
Secondary school	**5**	**13**		
Attended college or higher	**25**	**19**		
Substance use patterns				
Ethanol intake (mean of last 90 days), g/day	**6.2 (0.9)**	**203.6 (27.8)**	***t*_82_ = 5.980**	**<0.001**
ADS (total score)	**2.1 (0.4)**	**15.4 (0.9)**	***t*_82_ = 11.055**	**<0.001**
Smoking				
No. of participants responding yes/no	**4:31**	**32:17**	**Χ^2^_1_ = 24.200**	**<0.001**
FTND total score, mean (SE)	**4.8 (1.9)**	**6.1 (2.1)**	***t*_35_ = 1.123**	**0.269**

### Chronic ethanol consumption decreases tortuosity in the extracellular compartment

**Fig. 2:**
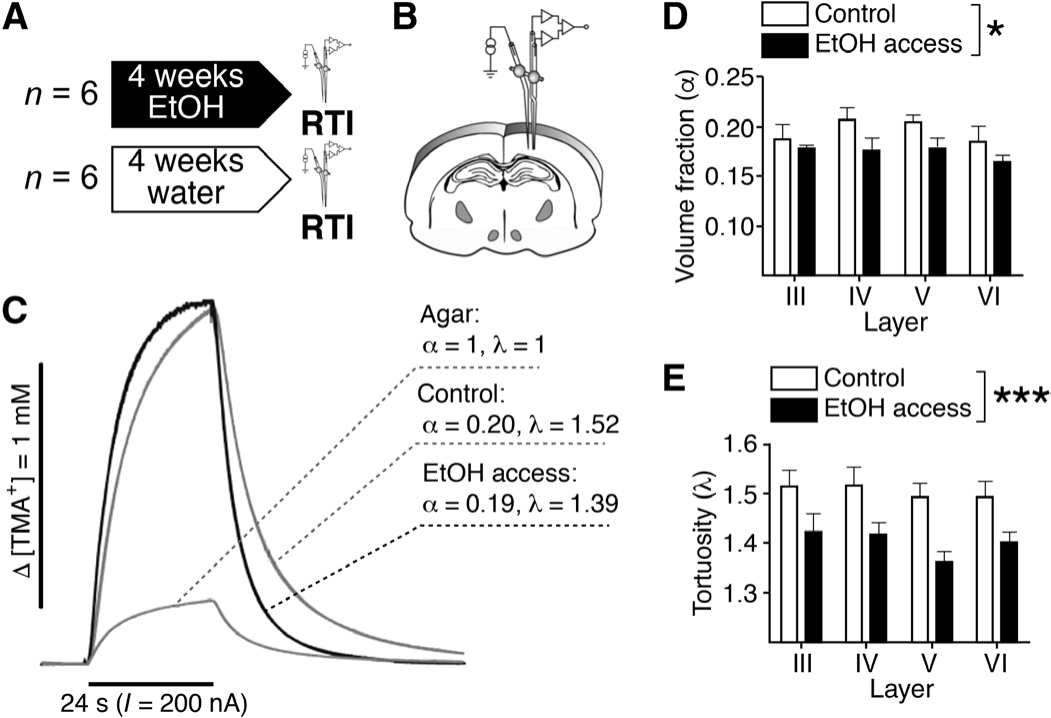
Alcohol alters the ECS geometry. (**A**) Experimental design. (**B**) Scheme illustrating the arrangement of electrodes in a typical experiment. (**C**) Representative diffusion curves and their parameters obtained in vivo in the cortical layer V of a control and an alcohol-exposed rat or in the diluted agar. (**D**) Quantification of volume fraction (α) in control (white) and alcohol-exposed (black) msP rats. A slightly significant effect of alcohol was found by two-way ANOVA [*F*(1,38) = 6.7, *P* < 0.05]. No effects of cortical layer or interaction between alcohol treatment and cortical layer were found. (**E**) Quantification of tortuosity (λ) in control (white) and alcohol-exposed (black) msP rats. A robust significant effect of alcohol was found by two-way ANOVA [*F*(1,38) = 21.6, *P* < 0.0001]. No effects of cortical layer or interaction were found. For all panels, *P < 0.05 and ***P < 0.001.

While MD provides brain-wide measures of water diffusion rate in the tissue, the contribution of different compartments (i.e., intracellular versus extracellular) to this measure cannot be disentangled. To test our hypothesis that alcohol may increase the diffusivity of released neurotransmitters in the ECS, we moved to real-time iontophoresis experiments. With this technique, the properties of the ECS can be precisely measured (*10*). More specifically, using two groups of animals, alcohol-drinking and naïve (*n* = 12, Fig. 2A), and by measuring the diffusion in the cortex of a tracer compound [the tetramethylammonium cation (TMA^+^)] injected into the ECS by a current pulse (Fig. 2B), we calculated the volume occupied by the ECS or the ECS volume fraction (α) and the geometrical factor tortuosity (λ; Fig. 2, D to E). Representative concentration time profiles in response to an iontophoretic pulse (TMA^+^ diffusion curves) are shown in Fig. 2C. Because no significant differences between cortical layers were found neither for α [*F*(3,38) = 1.06, *P* = 0.36; interaction, *F*(3,38) = 0.34, *P* = 0.80] nor λ values [*F*(3,38) = 0.8, *P* = 0.48; interaction, *F*(3,38) = 0.2, *P* = 0.92], the data from different layers were pooled for the statistical analysis. We found that 1-month alcohol drinking induced significant changes in the ECS properties (Fig. 2, D and E). A marginally significant decrease in the cortical ECS volume fraction [control, 0.196 ± 0.007; EtOH, 0.176 ± 0.004; *F*(1,38) = 6.7, *P* < 0.05] and a strongly significant decrease in the tortuosity [control, 1.503 ± 0.015; EtOH, 1.403 ± 0.015; *F*(1,38) = 21.6, *P* < 0.0001] were found in the alcohol-drinking group. These results are consistent with an effect of alcohol on the ECS that increases MD by eliminating barriers for diffusion. Overall, the robust reductions in tortuosity, together with the slight reduction in the ECS volume fraction, support the hypothesis of an alcohol-induced increase in volume transmission efficiency. Released neurotransmitters will be diluted less and reach farther in the ECS (see below).

### Down-regulation of microglial markers after chronic ethanol consumption

**Fig. 3:**
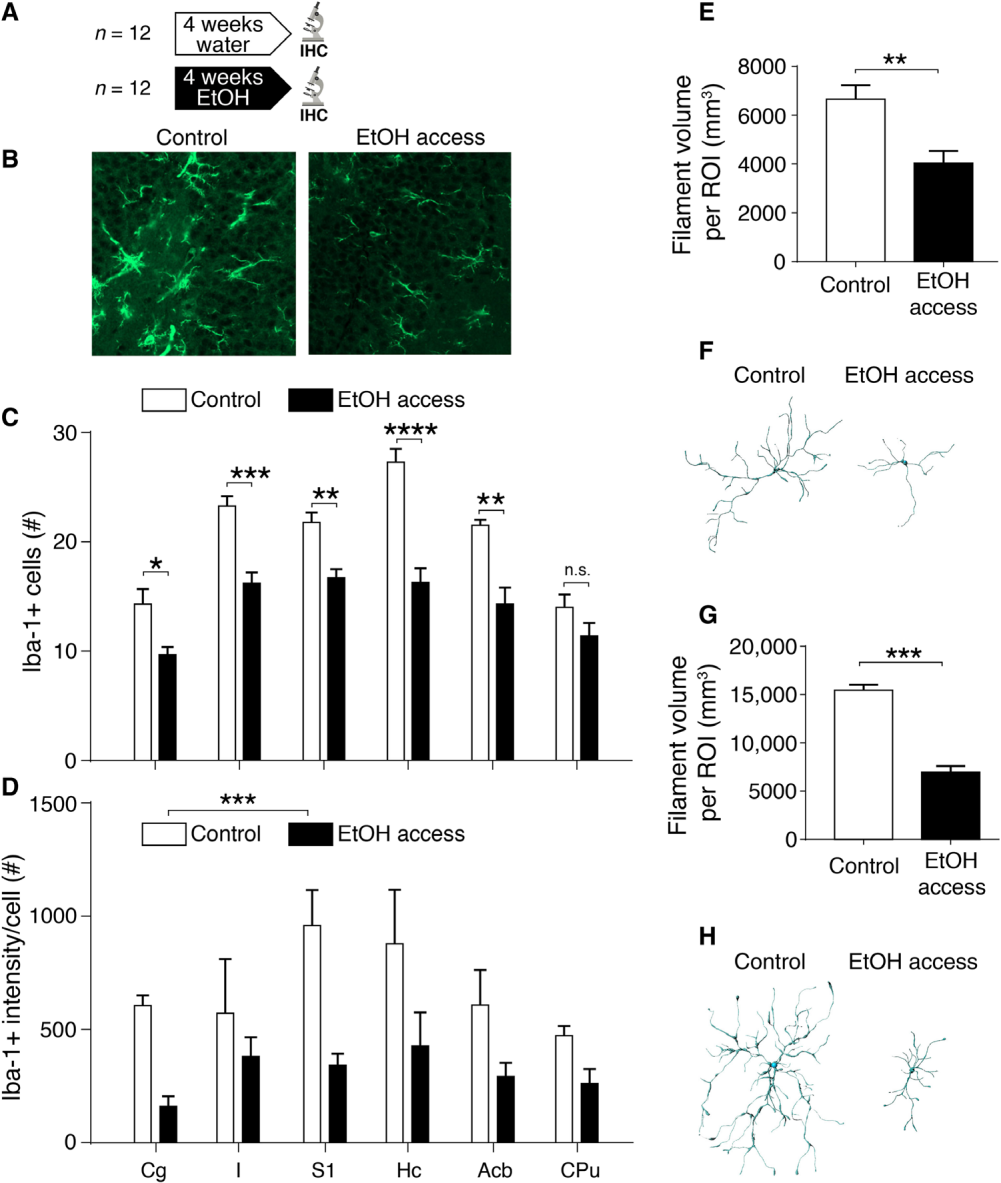
Microglial response to alcohol drinking. (**A**) Experimental design. IHC, immunohistochemistry. (**B**) Iba-1–positive (Iba-1+) immunostaining in histological sections from representative animals in the two experimental groups. Images were taken from the hippocampal formation (dentate gyrus). (**C**) Quantification of Iba-1+ cell numbers under control conditions (white) and after 1-month drinking (black). A robust significant effect of the experimental group was found by two-way ANOVA [*F*(1,40) = 97.7, *P* < 0.0001] and a significant interaction between factors [*F*(5,40) = 3.5, *P* = 0.01]. (**D**) Same as (C) but for staining intensity. A robust significant effect of the experimental group was found by two-way ANOVA [*F*(1,34) = 34.2, *P* < 0.0001]. No interaction was found [*F*(5,34) = 1.11, *P* = 0.38]. (**E**) Morphological analysis of 56 microglia cells in the nucleus accumbens, showing a reduced filament volume per ROI under EtOH conditions (unpaired *t* test, *t* = 6.1, df = 5, *P* = 0.0017). (**F**) Example of reconstructed microglia cells under the two conditions in the nucleus accumbens. (**G**) Morphological analysis of 56 microglia cells in the hippocampus, showing a reduced filament volume per ROI under EtOH conditions (unpaired *t* test, *t* = 14.8, df = 3, *P* = 0.0007). (**H**) Example of reconstructed microglia cells under the two conditions in the hippocampus. For all panels, *P < 0.05, **P < 0.01, and ***P < 0.001.

We next looked for histological correlates of the changes in diffusion parameters of the ECS. Typical changes in brain parenchyma affecting the ECS volume fraction and tortuosity include cell loss or proliferation, glial cell reactions (microglia and astrocytes), and changes in the composition of the extracellular matrix (*19*). Accordingly, we immunostained and quantified the staining intensity of astrocytes [glial fibrillary acidic protein (GFAP)], microglia (Iba-1), and chondroitin sulfate proteoglycan (CSPG); counted the number of both glial cell types; and further analyzed the morphology of astrocytes and microglial cells in histological sections obtained from two groups of msP animals, before and after 1 month of alcohol drinking (Fig. 3A). Knowing that MD changes induced by alcohol drinking persist into abstinence, we included a third group of msP rats in which 1 week of abstinence was forced after the drinking period (fig. S2, C and D). We focused the analysis in all ROIs used in the MRI analysis, when possible. From all the above-mentioned histological markers, microglial cells showed the strongest alcohol-induced response (Fig. 3B). The CSPG staining intensity demonstrated no significant changes between experimental conditions [two-way analysis of variance (ANOVA); *F*(1,68) = 0.19, *P* = 0.67; fig. S3], as was the case for the number of astrocytes [two-way ANOVA; *F*(1,110) = 2.2, *P* = 0.14; fig. S3] and their volume [two-way ANOVA; *F*(1,110) = 0.54, *P* = 0.46; fig. S3]. However, for Iba-1 staining, we found a strong alcohol-induced decrease in microglial cell numbers [Fig. 3C; two-way ANOVA; *F*(1,40) = 97.7, *P* < 0.0001] and staining intensity after 1 month of alcohol drinking [Fig. 3D; two-way ANOVA; *F*(1,34) = 34.2, *P* < 0.0001]. During the abstinence period, the numbers of microglial cells partially recovered (fig. S2C), but not the staining intensity (fig. S2D). Morphological reconstruction of microglial cells under different conditions showed a significant decrease in the filament volume per ROI in both nucleus accumbens and hippocampus (Fig. 3, E to H; unpaired *t* test, *t* = 6.1, df = 5, *P* = 0.0017; unpaired *t* test, *t* = 14.8, df = 3, *P* = 0.0007, respectively), which does not reverse under abstinence conditions (unpaired *t* test, *t* = 1.454, df = 8, *P* = 0.1840; unpaired *t* test, *t* = 0.3288, df = 4, *P* = 0.75, respectively). We further analyzed the neuronal cell viability counting the number of neurons (NeuN) and the staining intensity (NeuN and neurofilament; fig. S3).

Overall, the quantitative histological result agrees with brain MD and ECS tortuosity changes in our model of moderate alcohol drinking. For ECS properties, a reduction in microglial cell number and ramifications in the tissue means a reduction of diffusion barriers, which is consistent with a decrease in ECS tortuosity and an increase in MD. We then tested whether the microglial population could account for the observed magnitude of MD change.

### Reducing microglial content or complexity in gray matter enhances MD

**Fig. 4:**
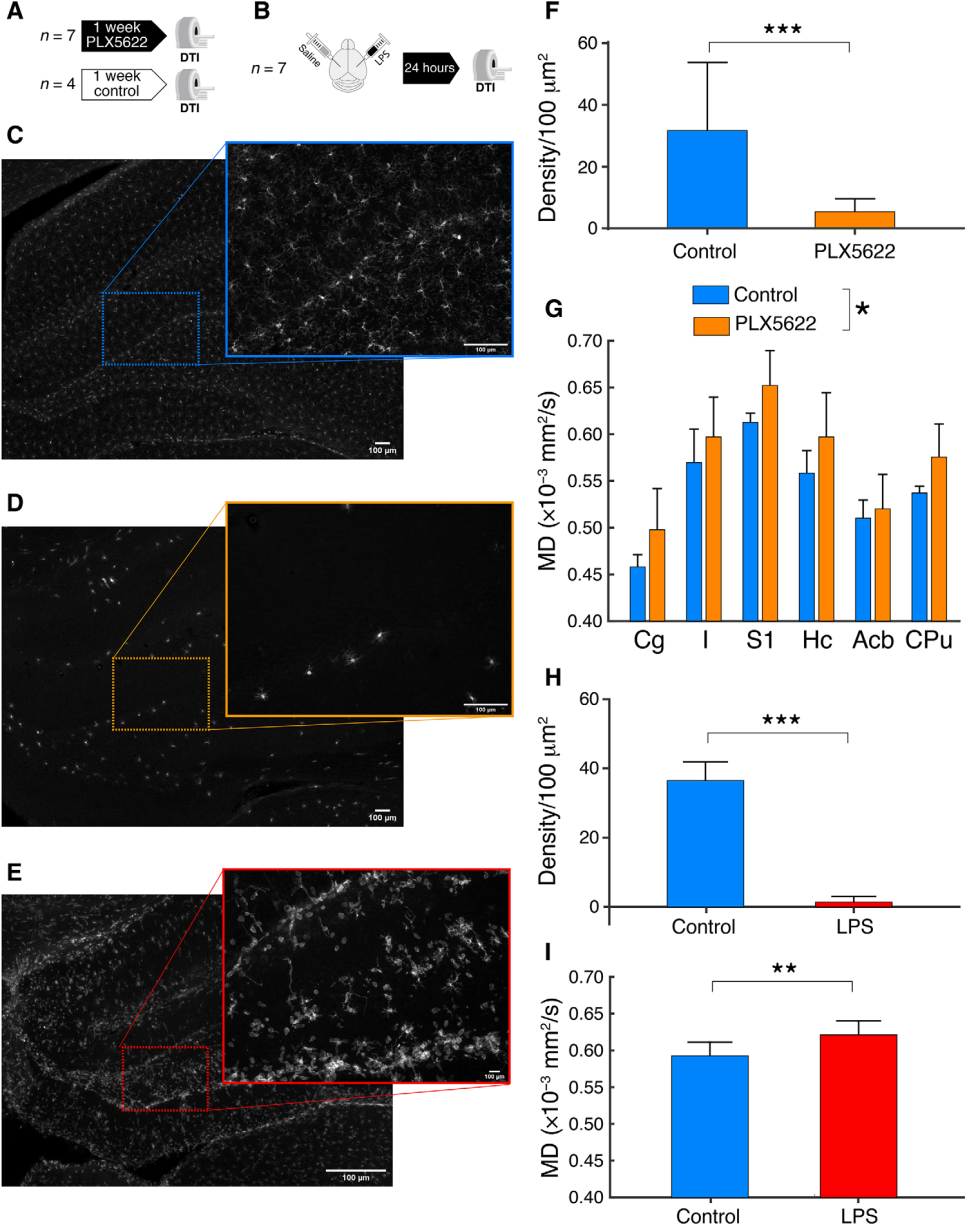
MD increases following directed microglia interventions. (**A** and **B**) Experimental designs for PLX5622 and lipopolysaccharide (LPS), respectively. (**C**) Representative Iba-1 staining in the hippocampus of a control rat. (**D**) Representative Iba-1 staining in the hippocampus after 7 days of PLX5622 administration. (**E**) Representative Iba-1 staining of an LPS-injected hippocampus 24 hours after surgery. (**F**) Quantification of microglial processes density for control rats and PLX5622 (unpaired *t* test, *t* = 7.6, df = 467, *P* < 0.0001). (**G**) MD measured in six gray matter ROIs for control rats and rats treated with PLX5622. *F*(1,8) = 6.3, *P* = 0.03. (**H**) Reduction of microglial processes density in the LPS-injected hemisphere versus control (unpaired *t* test, *t* = 69.11, df = 237, *P* < 0.0001). (**I**) Increase MD in the LPS-injected hemisphere versus control (paired *t* test, *t* = 3.7, df = 6, *P* = 0.009). *P < 0.05, **P < 0.01, and ***P < 0.001. Cg, cingulate cortex; I, insular cortex; S1, primary somatosensory cortex; Hc, hippocampus; Acb, accumbens; CPu, caudate-putamen.

To demonstrate the sufficiency of the microglial response to cause the observed increase in MD by alcohol, we reduced the density of microglia processes in the gray matter by two alternative approaches. First, we used the selective colony stimulating factor 1 receptor (CSF1R) inhibitor PLX5622 (Plexxikon Inc.), which causes an extensive and specific reduction of microglial cell numbers and complexity (*20*). As expected, systemic administration of PLX5622 (7 days; see Materials and Methods) significantly reduced the microglial cell population in the brain (Fig. 4, D and F). Microglial depletion was associated with a significant increase in MD, as measured by DTI [*F*(1,8) = 6.3, *P* = 0.03; Fig. 4G].

In a second experiment, we induced a focal microglial reaction by injecting lipopolysaccharide (LPS), an endotoxin composed of cell wall components of bacteria, known to cause a robust activation of microglia characterized by a thickening of cell bodies and a strong reduction of its ramifications, resulting in decreased complexity (*21*). Stereotaxic injection of LPS in one hippocampal hemisphere, with the contralateral receiving saline and serving as intra-animal control, produced the expected alteration in microglia morphology (Fig. 4H) and a significant increase in MD selectively in the LPS-injected hemisphere [*t*(6) = 3.7, paired, *P* < 0.01; Fig. 4I].

In both experiments, the reduction in microglial markers was linked to an increased MD of comparable magnitude, as observed after chronic alcohol exposure (alcohol, 5.6%; PLX5622, 6.5%; LPS, 4.9% versus respective controls).

### Modeling dopamine diffusion for different ECS geometries

**Fig. 5:**
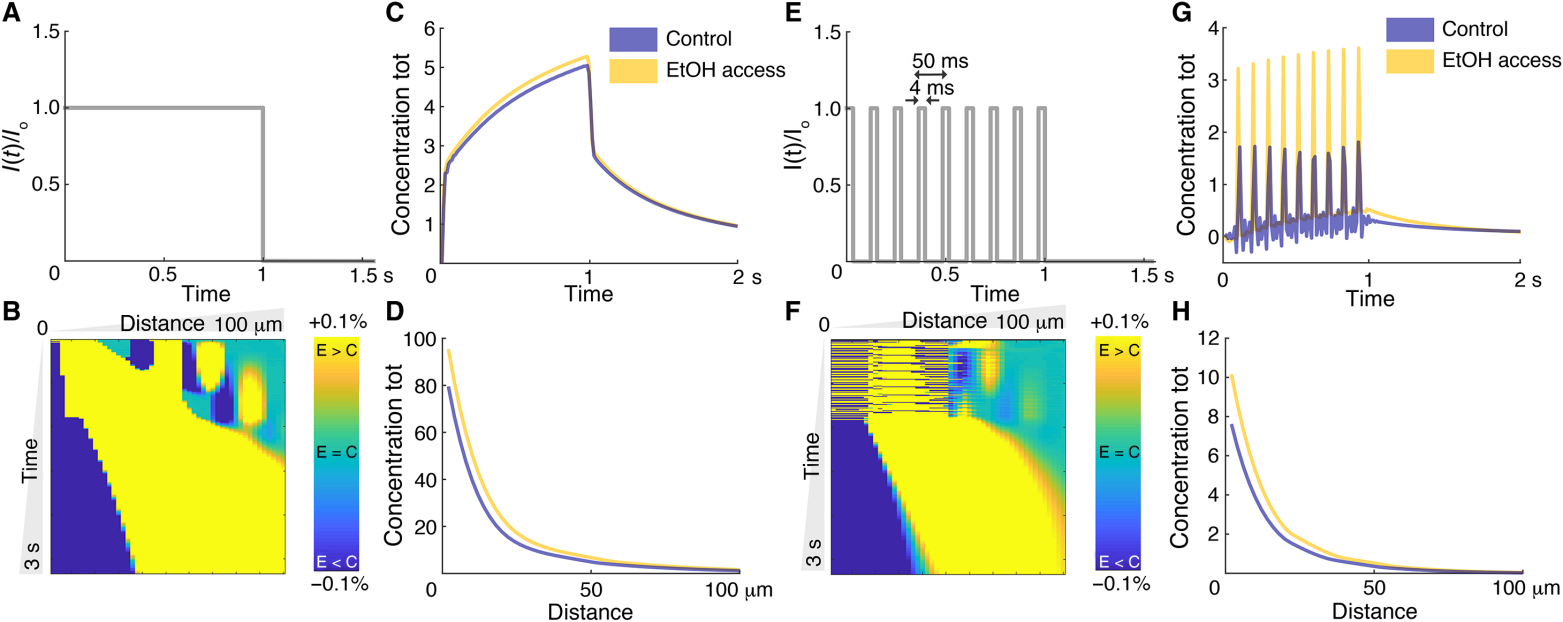
Dopamine concentration across space and time for alcohol and control conditions. (**A**) Tonic dopamine release pattern (flat). (**B**) Difference between dopamine concentrations in EtOH and control conditions in the range of 1 to 100 μm and 0 to 3 s. Yellow indicates EtOH > control, and dark blue indicates control > EtOH. (**C**) Total (tot) concentration integrated across all the space, for each time and for the two conditions. (**D**) Total concentration integrated across all times, for each spatial position and for the two conditions. (**E**) Phasic dopamine release pattern normalized (burst of 4-ms width and frequency of 20 Hz). (**F**) Difference between dopamine concentrations in EtOH and control conditions in the range of 1 to 100 μm and 0 to 3 s. Yellow indicates EtOH > control, and dark blue indicates control > EtOH. (**G**) Total concentration integrated across all the space, for each time and for the two conditions. (**H**) Total concentration integrated across all times, for each spatial position and for the two conditions.

We next numerically solved the diffusion equation for the two ECS geometries described in our experiments, one for the alcohol conditions and one for the control, according to the formula reported by Nicholson (*17*). This equation also takes into account dopamine uptake through the dopamine transporter (DAT), characterized by a maximum velocity (Vm) and a Michaelis-Menten constant (see Materials and Methods). As expected, the alterations found in tortuosity and the ECS volume fraction had an impact on the spatial and temporal dynamics of the dopamine concentration (Fig. 5). We implemented two modes of dopamine release, tonic and phasic. For both dopamine-release patterns, dopamine was, on average, more available in the ECS of alcohol-exposed subjects compared to controls, especially at shorter and intermediate times. Although the evidence is not robust across studies, there were previous indications that alcohol could interfere with the DAT kinetics (*22*). While Budygin *et al.* (*23*) found effects of alcohol on DAT Vm, others failed to find it (*24*) or found mixed results (*25*). Nevertheless, we decided to consider this possibility by using a broad range of Vm values, covering those found in the above literature (Vm = −60 to 30%, +30 and +60% versus the baseline value). The results shown in fig. S4 demonstrated that our findings on the spatiotemporal evolution of dopamine concentration were robust across the range of Vm values tested.

## DISCUSSION

In this work, we used diffusion properties to characterize the brain’s gray matter microstructure in AUD. Using noninvasive DTI in both alcohol-drinking rats and patients with AUD, we reported remarkably similar patterns of increased MD by alcohol, which persist into early abstinence. We then investigated the mechanism underlying this translational MRI observation and found a significant decrease in the tortuosity of the ECS, which could be explained by a microglial reaction under alcohol conditions. We studied how the altered ECS geometry affects the diffusion dynamics of released neurotransmitters and propose it as part of the mechanism supporting the potent addictive effect of alcohol.

The first key finding of this study is the notably similar pattern of increased MD in the brain parenchyma of human alcoholics and alcohol-drinking rats. In a recent classification experiment using multimodal brain imaging to separate different stages in msP rats exposed to alcohol, we identified MD as an important feature that contributes to the high performance of the machine-learning classifier (*6*). The new finding, in human patients, reinforces the relevance of gray matter MD as an important microstructural property affected by AUD and highlights the value of DTI applied to gray matter, in addition to white matter, as commonly done, as a source of imaging biomarkers. A previous study investigating verbal episodic memory in human alcoholics also reported an association between increased apparent diffusion coefficient (i.e., the diffusivity along one direction) in the frontal and temporal lobe of alcoholic patients, but not volume, with a decrease in memory performance (*26*). To our knowledge, MD changes in gray matter of alcoholics have not been studied so far. Convergent results in both species were found in our study, disregarding confounding factors in the human population, such as co-abuse or comorbidities, absent in the controlled animal model. Moreover, the long-time trajectory of human AUD, typically more than 10 years, may not be required for developing MD increase because 1 month of drinking in rats suffices to induce the phenomenon, suggesting its contribution to the early processes underlying alcohol-induced brain transformations. The translation validity of this animal model has been extensively validated by behavioral, molecular, and functional evidence (*4*, *5*). This new finding complements recent observations in the white matter of alcoholic patients and rats, in which convergent DTI alterations were found in both species (*4*), and underlines the important role that animal-based assays can play in overcoming the translational crisis in psychiatric research.

While DTI provides easily accessible noninvasive indicators of tissue abnormalities, the interpretation of the observed findings in terms of the cellular substrate underlying the changes is challenging, because several biological processes may cause similar changes in diffusion properties, especially in gray matter (*27*–*28*). Hence, MD reflects the average diffusion from all compartments present in the imaging voxel and does not allow precise differentiation between cellular and extracellular compartments. Thus, by using real-time iontophoresis, we specifically focused on the geometry of the ECS. Our results, demonstrating a decrease in the tortuosity of the ECS in alcohol-drinking rats, provided the physical substrate for the observed MD increase in both species. We went further to support the MRI and real-time iontophoresis findings with a histological analysis in rats and found a strong association between microglia and the increased tissue diffusivity. Alcohol drinking induced a reduction in the number of microglial cells and their complexity, measured as the ramification pattern of their processes. We demonstrate that both reductions in microglia complexity or cell numbers generate by itself a robust increase in gray matter MD, supporting the capacity of microglia alterations to provoke the observed MD changes in AUD. The results are summarized in fig. S5. In good agreement, several studies in postmortem human alcoholics (*29*–*31*) and rats (*32*) found a reduction of glial density, although some studies reported increased expression of the microglia marker Iba-1 in the brains of alcoholic individuals and rats [recently reviewed in (*8*)]. Notably, neuroinflammation has been proposed as a mechanism of alcohol-related brain damage (*33*), for example, via epigenetic modulation of microglia-specific gene expression (*34*–*35*), and microglial cells to be critical regulators of alcohol responses in the brain (*36*). Pharmacological manipulations of microglial cells in otherwise control rats, using LPS or PLX, generated an MD increase of comparable magnitude to that induced by alcohol exposure. LPS administration has been associated with increased alcohol consumption in rodents (*37*).

White matter DTI alterations in alcoholic patients progress in early abstinence (*4*). This unexpected finding was interpreted as an indication of an underlying process triggered by alcohol that progresses after drinking cessation. In contrast, gray matter shows no detectable progression of the DTI signal during early abstinence. This is corroborated by our immunohistochemical analysis in rats that demonstrated reduced Iba-1 expression for at least 1 week after cessation of alcohol. The differential glia responses in gray and white matters emerge as important factors in the pathology of AUD, particularly in the early abstinence phase when patients are highly vulnerable to relapse and decisions about optimal treatment choices have to be made. Once the alcohol-related microglia response is understood and can be interfered with, DTI may provide easily accessible biomarkers for detection of early warning signs or guiding individualized therapies.

We took advantage of well-developed diffusion equations for dopamine neurotransmission (*17*) to theoretically test the impact of the experimentally measured changes in the ECS on the extracellular dopamine concentration in time and space. These analytical calculations support the hypothesis that facilitated diffusivity of dopamine (and other small molecules) due to altered ECS could progressively enhance their signaling properties. Meta-analyses demonstrate a modest increase of dopamine (and other monoamines) release in response to ethanol (*38*, *39*). The magnitude of dopamine increase is unlikely to explain the addictive properties of alcohol in some users. It is intriguing to speculate that a synergistic combination of a primarily weak reinforcer with progressively enhanced volume neurotransmission due to increased extracellular diffusivity might comprise a previously unidentified mechanism to explain the slow onset but potent addictive effect of alcohol. Support for this hypothesis awaits further experimental demonstration.

Additional mechanistic implications are also possible. First, fluid-transfer pathways that subserve clearance of waste products and metabolites through the glymphatic system could be affected (*38*). Recently, a differential effect of alcohol in the brain clearance was found in chronically alcohol-injected mice, with low alcohol doses increasing and medium to high levels reducing the glymphatic system efficiency (*39*). However, these effects are largely dependent on astroglia, and the main effect observed here concerns the microglia, whose importance for ECS shape is well established (*40*). Second, some microglia fraction is also important for structural plasticity, i.e., synapse pruning and formation and ultimately, learning (*40*). The reduction in the microglia pool and ramification pattern could thus promote the formation of stable or more rigid memories. These effects may interact with the here-reported increased diffusion range and enhanced volume neurotransmission, which together may result in a specific form of functional plasticity. Increased neurotransmitter concentration over time of, e.g., dopamine, glutamate, or neuropeptides combined with reduced synapse turnover may interact for turning alcohol’s weak rewarding properties into the powerful habit-forming effects that eventually lead to addiction in some people. To understand and ultimately reverse these changes may aid the development of more effective treatment interventions.

## MATERIALS AND METHODS

### Animal study

All animal experiments were approved by the Animal Care and Use Committee of the Instituto de Neurociencias de Alicante, Alicante, Spain, and comply with the Spanish (law 32/2007) and European regulations (EU directive 86/609, EU decree 2001-486, and EU recommendation 2007/526/EC).

In total, 84 male msP rats were used. The line was created by selective breeding for high voluntary alcohol consumption (*6*, *41*). Rats were imported to the animal facilities in Alicante (MRI experiments, *n* = 36) and in Prague (real-time iontophoresis experiments, *n* = 12) from the breeding facility at the School of Pharmacy, University of Camerino, Italy, at an age of 8 weeks. Animals for the immunohistochemistry study were prepared and directly exposed to alcohol in Camerino. Rats were individually housed under controlled temperature (22° ± 2°C) and relative humidity (55 ± 10%) on a 12-hour light/12-hour dark cycle in transparent polycarbonate cages with bedding material. A wooden stick and nesting material were given as enrichment. All experiments were performed in accordance with Spanish (law 32/2007), Czech Republic (Animal Care Committee approval number 149/2010), and European regulations (EU directive 86/609 and EU decree 2001-486). Rats were given 30 days of access to two drinking bottles, one containing water and the other 10% (v/v) EtOH in water. Control rats were kept under same conditions but were given access to water only. Fluid consumption and animal weight were registered every 2 to 3 days, concomitant with replacement of the bottles’ content. After 1 month of two-bottle free-choice drinking regime, the EtOH-containing bottle was removed. For some experiments, rats were kept for another week without EtOH access. See Figs. 1 (A and B), 2A, and 3A for experimental designs.

### Human study

The participants were 84 men enrolled in two different groups: (i) a cohort of 35 healthy controls and (ii) 49 treatment-seeking patients with AUD scanned at two time points (at 1 week after admission into the clinic and completion of detoxification treatment and after 2 to 3 weeks). The study was conducted at the Central Institute for Mental Health in Mannheim, Germany. The local ethics committee approved study procedures in accordance with the Declaration of Helsinki (*42*). Participants gave written consent and did not receive any stipend. Inclusion criteria and assessment are reported in the Supplementary Materials. Descriptive statistics of demographic data and clinical descriptors appear in Table 1.

### MRI experiment

Human scanning was performed with a 3-T whole-body tomograph [MAGNETOM Trio with total imaging matrix technology; Siemens, Erlangen, Germany]. DTI data were acquired using an echo-planar imaging spin-echo diffusion sequence with the following parameters: repetition time (TR) = 14 ms, echo time (TE) = 84 ms, 41 gradient orientations uniformly distributed plus one nondiffusion weighted images, *b* value = 1000 s/mm^2^, matrix size = 128 × 128 × 64, and isotropic resolution of 2 mm^3^.

MRI experiments on rats were performed on a 7-T scanner (Bruker, Biospec 70/30, Ettlingen, Germany) using a receive-only phase array coil with integrated combiner and preamplifier in combination with an actively detuned transmit-only resonator. DTI data were acquired using an echo-planar imaging diffusion sequence, with 30 uniform distributed gradient directions, *b* = 670 s/mm^2^, four nondiffusion-weighted images, TR = 4000 ms, and TE = 23 ms. Fourteen horizontal slices were set up to field of view = 32 mm by 32 mm, matrix size = 128 × 128, in-plane resolution = 0.25 mm by 0.25 mm, and slice thickness = 1 mm.

MRI datasets were processed as follows: DTI data were corrected for motion and eddy current distortions, and local diffusion tensor was fitted using ExploreDTI (*43*). From the diffusion tensor components, MD maps were extracted. MD maps were nonlinearly registered to high-resolution human and rat MD templates (*43*, *44*) using an advanced normalization approach (*44*). Voxel-wise comparisons were carried out to identify alcohol- and abstinence-induced differences. For the cross-sectional analysis, a general linear model was used within a voxel-wise, permutation-based, and nonparametric statistical framework (*45*) to test for significant differences controlling for age and multiple comparisons across clusters using threshold-free cluster enhancement. For the longitudinal design, the statistic was applied on the difference between time points. All statistical analysis was performed on the gray matter masks only. For both rats and humans, a gray matter segmentation was available in the template, and only voxels with a probability of >90% belonging to gray matter were used. Voxels containing cerebrospinal fluid (MD > 2 × 10^−3^ mm^2^/s) were also excluded from the statistics to avoid contamination.

In addition, for direct comparison with histological and real-time iontophoresis experiments, an ROI-wise analysis was also performed for the nucleus accumbens, caudate-putamen, cingulate cortex, hippocampus, insular cortex, and primary somatosensory cortex. In rats, ROIs were calculated using a study-based template, according to (*6*). To test for alcohol effect in AUD humans versus control, a one-way repeated-measures ANOVA was used with age as a covariate and group (AUD at first scan and healthy control) as the between-subject factor. Similarly, longitudinal differences in MD between AUD at first scan and 2 to 3 weeks after were tested using repeated-measures ANOVA, with time as the within-subject factor. Post hoc tests were corrected for multiple comparisons using false discovery rate.

To test the alcohol effect in msP rats, a two-way repeated-measures ANOVA was conducted. Time (baseline and 1 month of ethanol consumption) was defined as the within-subject factor and group (control and alcohol-exposed rats) as the between-subject factor. To test the effect of abstinence, one-way repeated-measures ANOVA was conducted. Conditions were naïve, EtOH consumption, and abstinence for each of the six ROIs. If the assumption of sphericity was violated, then degrees of freedom of the *F* distribution were Greenhouse-Geisser corrected. If required, then post hoc tests were adjusted by Tukey-Kramer corrections.

### In vivo diffusion experiment with real-time iontophoresis

Animals were anesthetized with isoflurane (1.5% in a gas mixture of 35% O_2_/65% N_2_O) administered by a face mask, and their heads were fixed in a stereotaxic holder. Body temperature was maintained at 37°C with a heating pad. The somatosensory cortex was partially exposed by a burr hole of 1.5-mm caudal from bregma and 1.5-mm lateral from the midline, and the dura was carefully removed. The exposed brain tissue was bathed in warm (37°C) artificial cerebrospinal fluid containing 117 mM NaCl, 3 mM KCl, 35 mM NaHCO_3_, 1.25 mM Na_2_HPO_4_, 1.3 mM MgCl_2_, 1.5 mM CaCl_2_, 10 mM glucose, and 0.1 mM TMA^+^ (pH 7.4, 300 mOsM).

To determine the ECS diffusion parameters, that is, ECS volume fraction α (α = ECS/total tissue volume), tortuosity λ (λ^2^ = *D*/ADC, where *D* is the free diffusion coefficient, and ADC is the apparent diffusion coefficient in the brain tissue), and nonspecific uptake *k*′, we used the real-time iontophoretic method, developed by Nicholson and Phillips (*10*). Briefly, an extracellular marker such as the tetramethylammonium ion (TMA^+^, *M*_w_ = 74.1 Da) is administered into the ECS by an iontophoretic pulse, and the local time-dependent changes in concentration of TMA^+^ are measured with a TMA^+^ ion-selective microelectrode (TMA^+^-ISM). TMA^+^-ISMs were prepared by a procedure described in detail previously (*10*). Double-barreled TMA^+^-ISMs consisted of an ion-sensitive and a reference barrel, where the tip of the ion-sensitive one was filled with a liquid ion exchanger for K^+^ [Corning 477317 or IE 190 from World Precision Instruments, RRID:SCR_008593) that is highly sensitive to TMA^+^ ions; the rest of the barrel was backfilled with 150 mM TMA^+^ chloride. The reference barrel contained 150 mM NaCl. The electrodes were calibrated using the fixed-interference method before each experiment in a series of solutions of 150 mM NaCl + 3 mM KCl, with the addition of the following concentrations of TMA chloride: 0.25, 0.5, 1, 2, 4, 8, and 16 mM.

An electrode array was made by gluing a TMA^+^-ISM to an iontophoretic micropipette with a tip separation of 100 to 150 μm. Typical iontophoresis parameters were 20-nA bias current (continuously applied to maintain a constant transport number) and a +180-nA current step, with a 24-s duration to generate the diffusion curve. TMA^+^ diffusion curves were first recorded in 0.3% agar (Sigma-Aldrich, Steinheim, Germany) dissolved in a solution of 150 mM NaCl, 3 mM KCl, and 1 mM TMACl, in which diffusion is free and by definition, α = 1, λ = 1, and *k*′ = 0. The diffusion curves obtained in agar were analyzed to yield the electrode transport number (*n*) and the free-TMA^+^ diffusion coefficient (*D*) by a nonlinear curve-fitting algorithm with a modified diffusion equation using the VOLTORO program (*10*). Knowing *n* and *D*, the values of α, λ, and *k*′ can be obtained from the diffusion curves when the measurement is repeated in the tissue.

Diffusion measurements were performed in the mediolateral axis in depths of 400 to 2000 μm from the surface, in 200-μm intervals. The cortex is optimal because the chances that the pipette gets obstructed are much smaller compared to deeper brain structures, resulting in more robust measurements. Three diffusion curves were acquired in each depth, and the yielded values of the ECS diffusion parameters were averaged to obtain a representative value for the specific depth. The values of α and λ for an individual cortical layer were then calculated from the data obtained in the appropriate depths: 400 and 600 μm, layer III; 800 μm, layer IV; 1000, 1200, and 1400 μm, layer V; 1600 and1800 μm, layer VI; and 2000 μm, white matter (*42*, *43*). Statistical comparisons were performed as above using two-way ANOVA with group (control and EtOH exposed) and cortical layer (III to VI) as factors. As no interlayer statistical differences were found either for α or λ values, the data obtained in the individual layers were pooled.

### Immunohistochemistry

The brains were fixed (24 hours) in buffered 4% paraformaldehyde (pH 7.4) and then stepwise saturated with sucrose (10, 20, and 30%) for cryoprotection. After freezing, 40-μm serial coronal sections were cut on a freezing sliding microtome (HM550, Microm International GmbH, Waldorf, Germany). The slices were first incubated in blocking solution, which contained 5% ChemiBLOCKER (Millipore, MA) and 0.2% Triton X-100 in 0.01 M phosphate-buffered saline. This blocking solution was also used as the diluent for the antisera. The slices were then incubated with the primary antibodies at 4°C overnight. As primary antibodies, we used mouse anti-GFAP antibody conjugated with Cyanine 3 (Cy3) (Sigma-Aldrich, catalog no. C9205), mouse anti–chondroitin sulfate antibody CS-56 (Abcam, catalog no. ab11570), and mouse anti–Iba-1/apoptosis-inducing factor 1 antibody (Merck Millipore, catalog no. MABN92). Along with the unconjugated antibodies, secondary anti-mouse immunoglobulin M (IgM) Cy3 (Merck Millipore, catalog no. AP128C; used for CS-56 staining) or goat anti-mouse IgG antibodies conjugated with molecular probe Alexa Fluor 488 (Molecular Probes, catalog no. A11029; used for Iba-1 staining) were applied for 2 hours. Slices were mounted using a Vectashield mounting medium (Vector Laboratories, catalog no. H-1000).

The tissue sections were then examined using an LSM 5 DUO spectral confocal microscope equipped with 40× oil objective (Carl Zeiss AG, Germany). Evaluation of differences among the animal groups in GFAP- and Iba-1–positive cell numbers, astrocyte morphology, expression of CSPG, and Iba-1 expression was performed in the somatosensory cortex (lamina V), cingulum, hippocampus (dentate gyrus), insular cortex, caudate-putamen, and nucleus accumbens. To quantify the morphological differences, the slices were scanned using the fixed setup conditions and laser power. Median signal intensity in arbitrary units was evaluated by LSM 5 Image Browser (Carl Zeiss AG, Germany; LSM Image Examiner) in each section (in both Iba-1– and CSPG-stained slices). Analysis of Iba-1– and GFAP-positive cells was performed using a Fiji image-processing package (*46*), a modification of ImageJ software. Morphological three-dimensional analysis and reconstruction of Iba-1–positive cells were performed using IMARIS software (Bitplane). Dendritic density refers to the normalization and comparison of dendrites’ amount in space among different kinds/conditions of cells, taking into account all kinds of branching processes within the cell with the following equation: [sum of the terminal orders + number of terminals] × [total dendritic length/number of primary dendrites]. Statistical comparisons were performed using two-way ANOVA with group (control and EtOH exposed) and ROIs (nucleus accumbens, caudate-putamen, cingulate cortex, hippocampus, insular cortex, and primary somatosensory cortex) as factors, and unpaired *t* test with group (control and EtOH exposed) in the case of morphological analysis for nucleus accumbens and hippocampus.

### Microglia depletion and activation

Microglia depletion was achieved by administering the CSF1R inhibitor PLX5622 (Plexxikon Inc.) to *n* = 7 rats in two ways: as a dietary supplement in standard chow at 1200 parts per million (Research Diets) and with intraperitoneal injection of 50 mg/kg in vehicle once a day. *n* = 4 control rats were given the same chow without enrichment and were injected intraperitoneally once a day with the same doses of vehicle. After 7 days, the rats underwent DTI and histology according to the protocols previously described.

Microglia activation was achieved in *n* = 7 rats by intracranial injection in the dorsal hippocampus (coordinates bregma of −3.8 mm, dorso-ventral of 3.0 mm, and 2 mm from midline in the left hemisphere) of 2 μl of saline and LPS at a concentration of 2.5 μg/μl. The opposite hemisphere was injected with saline. Because structures too close to the craniotomized skull will contain artefacts in the MRI data, producing poor measurements quality, we selected the hippocampus, which is far enough from the surface. After 24 hours from the LPS injection, the rats were scanned using a DTI protocol and perfused for histology, as previously described.

### Numerical solutions to the diffusion equation for dopamine

The diffusion equation incorporating uptake, characterized by a Vm, and a Michaelis-Menten constant (Km), was transformed to an integral equation and solved numerically for the dopamine concentration *C*, according to the method reported by Nicholson (*17*) using the software Mathematica v10 (Wolfram, Champaign, IL). Two different substrates were generated, according to the ECS volume fraction α and tortuosity λ measured by real-time iontophoresis: control and EtOH. Control substrate had α =0.20 and λ =1.54, while EtOH substrate had α = 0.19 and λ =1.39 (see real-time iontophoresis results). The value for the free-diffusion coefficient of dopamine was assumed to be *D*_free_ = 6.9 10^−6^ cm^2^/s, the duration of the dopamine release from the source was 1 s, the radius of the source was 2 μm, the source current was 100 nA, Km was 0.15 μM, and Vm was 0.2 μM/s. To mimic the two dopamine fire modalities (tonic or phasic), we used two different current regimes: flat and burst. In the first one, the current was constant during the 1-s period; in the second regime, the current was on in bursts of 4 ms each, with a frequency of 20 Hz (*47*). The equation was solved in the grid *R* = 0 to 100 μm and *T* = 0 to 3 s using the function NDsolveValue with the following parameters: MaxSteps, 4,000,000; MaxStepFraction, 1/20; StartingStepSize, 1 × 10^−11^; AccuracyGoal, 20; and PrecisionGoal, 10. Regarding the numerical approach, solving the differential equation describing dopamine diffusion and uptake in the ECS requires a finite-difference method and is thus subject to round off and truncation errors. To make these errors comparable for the two cases of interest (alcohol and control), the boundary conditions have been chosen according to each effective diffusion coefficient, defined as the dopamine diffusion coefficient divided by the squared tortuosity.

## Supplementary Material

aba0154_SM.pdf

## SUPPLEMENTARY MATERIALS

Supplementary material for this article is available at http://advances.sciencemag.org/cgi/content/full/6/26/eaba0154/DC1

## Appendix

View/request a protocol for this paper from *Bio-protocol*.
