# Supplementary material for: Chronic alcohol consumption alters extracellular space geometry and transmitter diffusion in the brain
Source: Sci Adv. 2020 Jun 24;6(26):eaba0154. doi: 10.1126/sciadv.aba0154 (PMC7314532; doi:10.1126/sciadv.aba0154)
Supplement: aba0154_SM.pdf [file aba0154_SM.pdf]

Science Advances
AAAS

advances.sciencemag.org/cgi/content/full/6/26/eaba0154/DC1

Supplementary Materials for

Chronic alcohol consumption alters extracellular space geometry and transmitter diffusion in the brain

Silvia De Santis, Alejandro Cosa-Linan, Raquel Garcia-Hernandez, Lesia Dmytrenko, Lydia Vargova, Ivan Vorisek, Serena Stopponi, Patrick Bach, Peter Kirsch, Falk Kiefer, Roberto Ciccocioppo, Eva Sykova, David Moratal, Wolfgang H. Sommer*, Santiago Canals*

*Corresponding author. Email: wolfgang.sommer@zi-mannheim.de (W.H.S.); scanals@umh.es (S.C.)

Published 24 June 2020, *Sci. Adv.* **6**, eaba0154 (2020)
DOI: 10.1126/sciadv.aba0154

This PDF file includes:

Figs. S1 to S5
Tables S1 and S2

Supplementary Materials

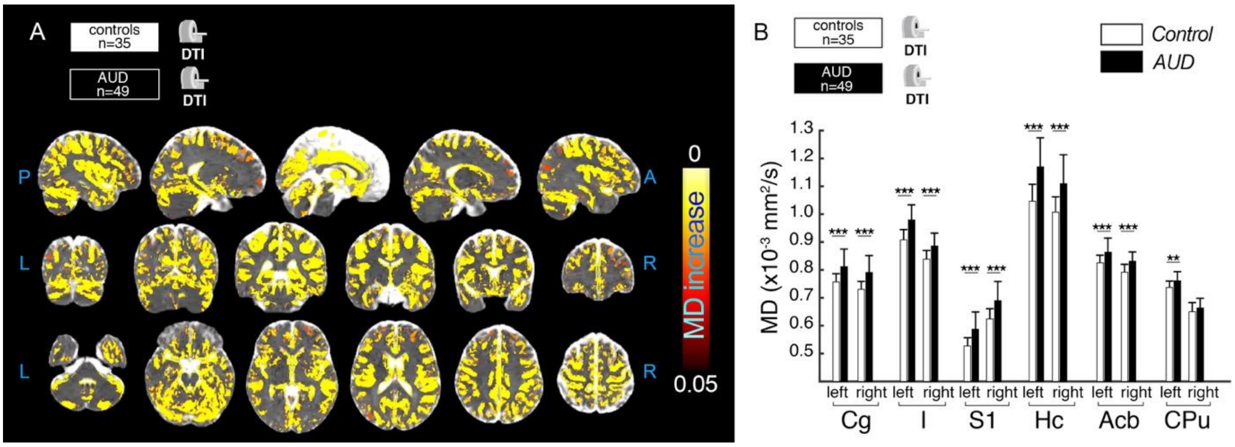

Figure S1. Effects of alcohol consumption in mean diffusivity for age-matched humans. A. Voxel-wise statistical analysis showing cross-sectional MD differences between controls (n=35) and a subset of age-matched alcohol-dependent patients at first scan (n=39). **B**. Mean MD values for controls and AUD patients at first scan. Reported statistics show cross-sectional differences between controls and AUD patients.

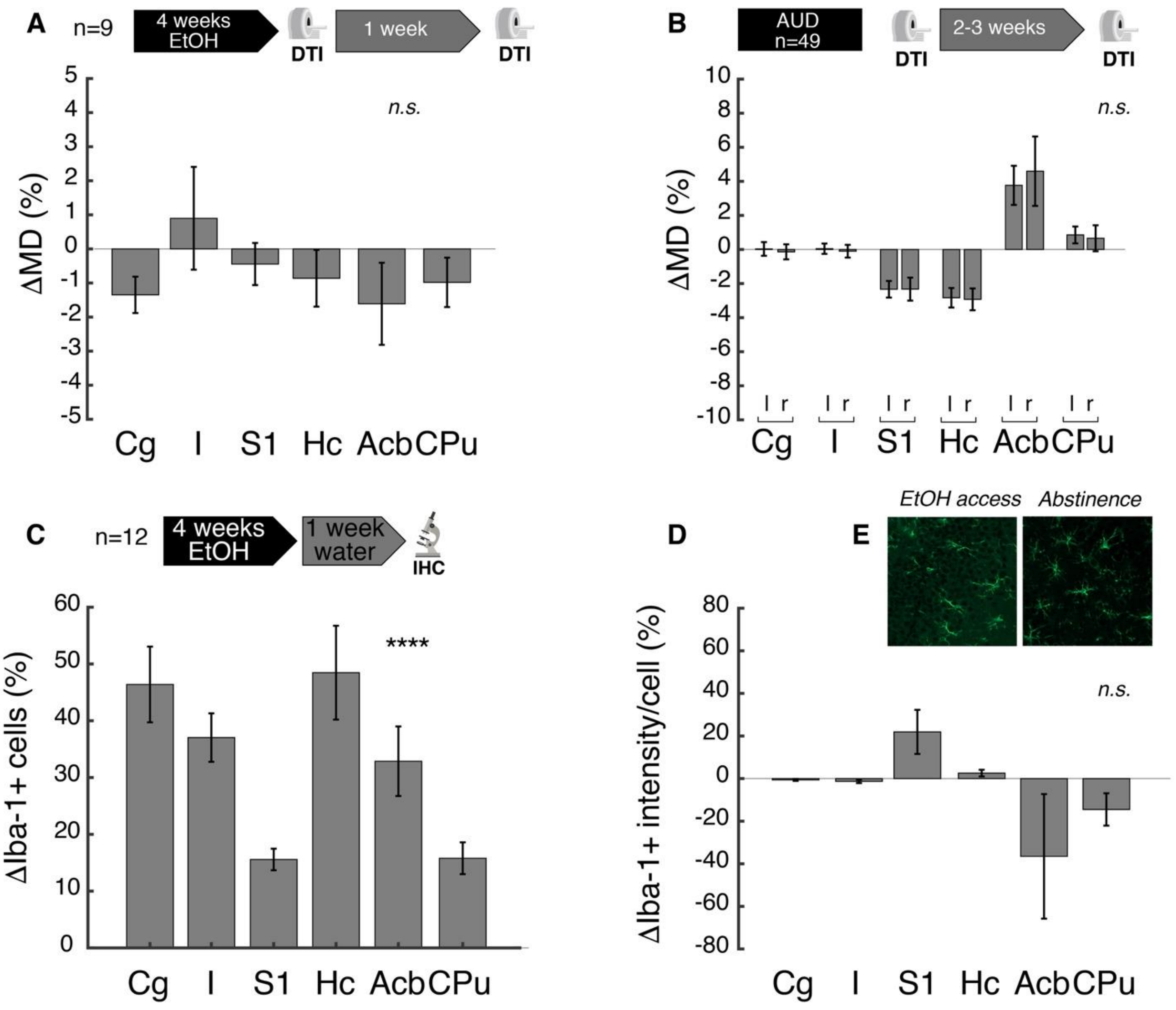

Figure S2. **Effects of abstinence in mean diffusivity and microglia markers.** MRI MD percent change in rats (**A**) and humans (**B**) in abstinence compared to alcohol stage. No differences were found between groups, indicating that MD remains enhanced during early abstinence. **C**. Change in number of Iba1+ cells in abstinence compared to alcohol stage in rats. There is a significant increase in the number of microglial cells in early abstinence ($F(1, 65)=58.5$, $p<0.0001$). No interaction was found ($F(5, 65)=2.29$, $p=0.06$). **D**. Same as (C) but for Iba1+ intensity. No statistically significant differences were found ($F(1, 62)=0.04$, $p=0.83$). **E**. Iba-1+ immunostaining in histological sections from representative animals in the two experimental groups. Images were taken from the hippocampal formation (dentate gyrus).

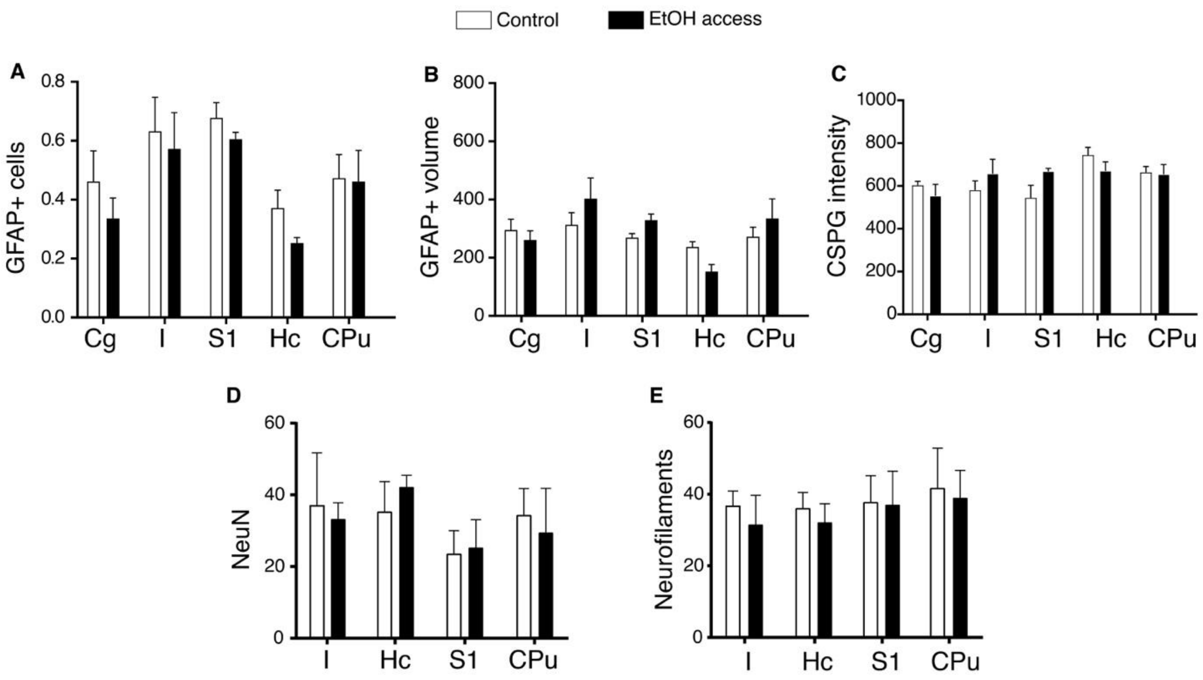

Figure S3. **Astroglial, CSPG and neuronal response to alcohol drinking.** **A**. Astroglial response to alcohol drinking measured as number of GFAP+ cells. **B**. Volume of GFAP+ cells in alcohol versus control animals. **C**. CSPG response to alcohol drinking and abstinence. **D**. NeuN staining in alcohol versus control animals. **E**. Neurofilaments staining in alcohol versus control animals. None of the staining is statistically different between control and exposed animals. *Cg: Cingulate cortex; I: insular cortex; S1: primary somatosensory cortex; Hc: hippocampus; CPu: caudate-putamen.*

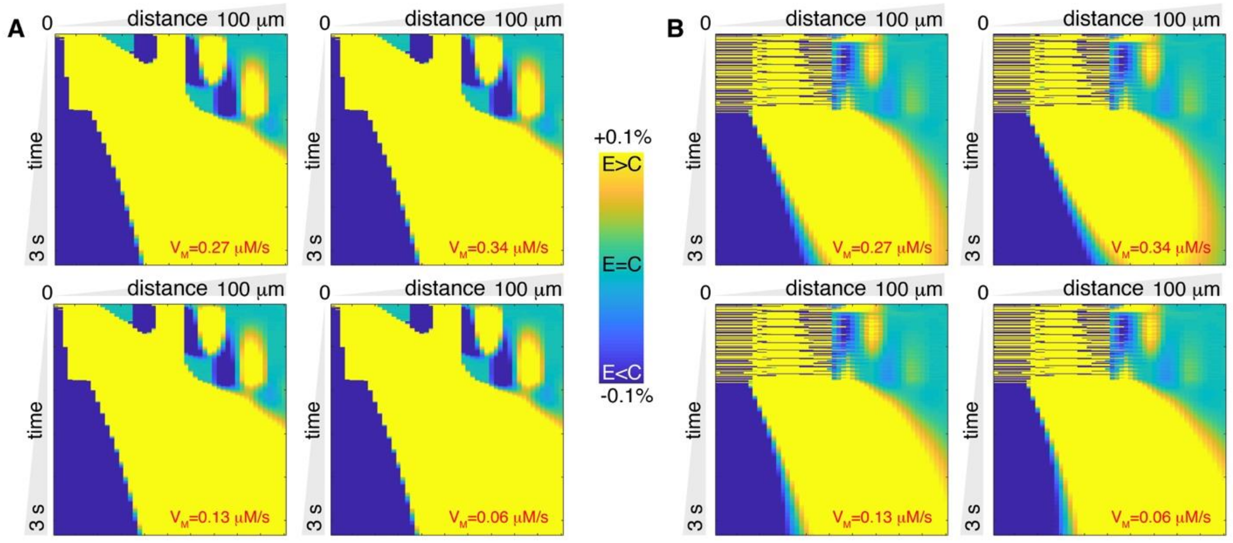

Figure S4. **Dopamine concentration for different velocities.** Difference between dopamine concentrations in EtOH and control conditions in the range 1-100 µm and 0-3 s for tonic (**A**) and phasic (**B**) dopamine release patterns. In the four panels, the parameter V_M is varied to reproduce values found experimentally.

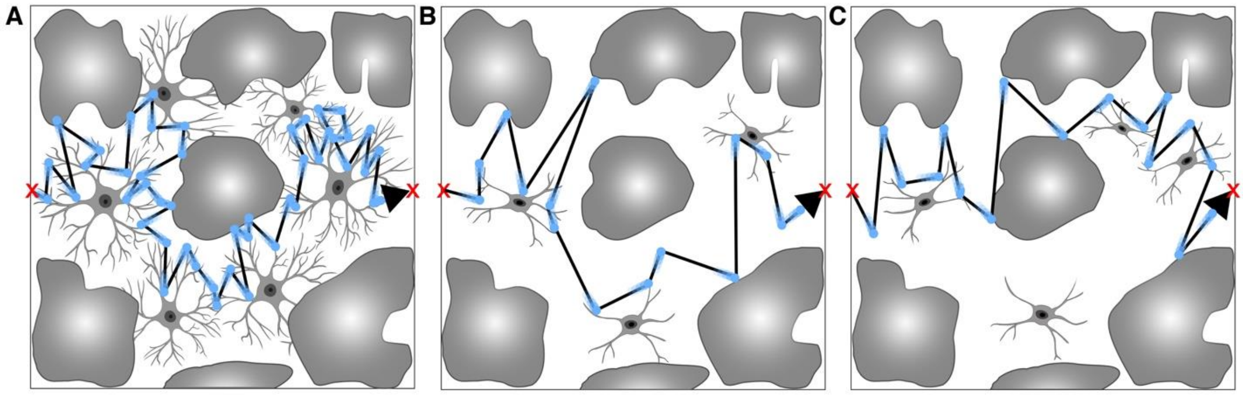

Figure S5. ECS in alcohol and abstinence. A. Schematic representation of random walk-like diffusion in ECS containing microglia and astrocyte in control condition. **B**. Diffusion in ECS compatible with alcohol condition, characterized by a reduced microglia density and less complex ramifications. **C**. Diffusion in ECS compatible with abstinence condition, where the density of microglia has slightly increased, but the complexity is still lower than controls.

Table S1. Effects of alcohol consumption in mean diffusivity (MD) in msP rats (n=18 with EtOH access and n=18 controls).

ROI	Interaction Group×Time		Group effect		Time effect		Mean diffusivity (mm²/s × 10³)	Baseline	1 month[1]
	$F_{(1,34)}$	p	$F_{(1,34)}$	p	$F_{(1,34)}$	p		Mean±SD	Mean±SD
Cg	21,6	0,000	35,3	0,000	1,5	0,219	Control	0,838±0,024	0,820±0,024*
							EtOH access	0,850±0,017	0,879±0,031*[,###]
I	38,0	0,000	0,0	0,859	38,0	0,081	Control	1,150±0,051	1,060±0,055***
							EtOH access	1,081±0,025[###]	1,149±0,049*[,#]
S1	38,0	0,000	1,4	0,247	38,0	0,017	Control	0,850±0,015	0,899±0,030***
							EtOH access	0,850±0,020[##]	0,920±0,025***[,#]
Hc	38,0	0,000	0,2	0,699	38,	0,103	Control	1,133±0,036	1,107±0,031*
							EtOH access	1,115±0,023	1,162±0,041***[,##]
Acb	13,9	0,001	32,2	0,000	2,7	0,106	Control	1,049±0,032	1,028±0,046
							EtOH access	1,070±0,048	1,121±0,046*[,###]
CPu	38,0	0,000	0,160	0,692	38,0	0,008	Control	0,899±0,026	0,874±0,028*
							EtOH access	0,863±0,019[###]	0,915±0,030***[,###]

[1]Significantly different from Baseline *p<0.05, **p<0.01, ***p<0.001; significantly different from control [#]p<0.05, [##]p<0.01, [###]p<0.001

Table S2. Effects of alcohol consumption and abstinence in mean diffusivity (MD) in humans.

Mean diffusivity (mm²/s × 10³)

ROI		$F_{(1,81)}$	P	Controls Mean±SD	AUD [1] Mean±SD
Cg1	left	23,5	<0,001	0,758±0,005	0,831±0,010***
	right	9,0	0,004	0,731±0,005	0,810±0,010**
I	left	29,5	<0,001	0,909±0,006	0,995±0,010***
	right	28,1	<0,001	0,839±0,005	0,704±0,011***
Cpu	left	10,8	0,001	0,738±0,004	0,772±0,006**
	right	16,7	<0,001	0,651±0,005	0,676±0,007***
Hc	left	14,6	<0,001	1,047±0,010	1,191±0,016***
	right	23,4	<0,001	1,008±0,009	1,127±0,015***
Acb	left	13,2	<0,001	0,826±0,005	0,870±0,007***
	right	12,8	0,001	0,792±0,005	0,836±0,005**
S1	left	25,0	<0,001	0,528±0,005	0,596±0,009***
	right	15,2	<0,001	0,808±0,007	0,884±0,010***

[1]Significantly different from Control *p<0.05, **p<0.01, ***p<0.001 corrected.
